# Palliative care in nursing training: higher education course coordinators’ perception

**DOI:** 10.1590/0034-7167-2022-0222

**Published:** 2023-10-09

**Authors:** Rafaella Guilherme Gonçalves, Luciane Paula Batista Araújo de Oliveira, Carlos Jordão de Assis Silva, Tatiana Maria Nóbrega Elias, Isadora Lorenna Alves Nogueira, Rejane Maria Paiva de Menezes

**Affiliations:** I Universidade Federal do Rio Grande do Norte. Natal, Rio Grande do Norte, Brazil

**Keywords:** Palliative Care, Professional Training, Education, Nursing, Higher Education Institutions, Teaching, Cuidados Paliativos, Capacitación Profesional, Educación en Enfermería, Instituciones de Enseñanza Superior, Enseñanza, Cuidados Paliativos, Capacitação Profissional, Educação em Enfermagem, Instituições de Ensino Superior, Ensino

## Abstract

**Objectives:**

to analyze undergraduate nursing course coordinators’ perception about nursing training in palliative care.

**Methods:**

a descriptive study, with a qualitative approach and thematic content analysis, carried out with coordinators of nursing courses in Higher Education Institutions in Rio Grande do Norte.

**Results:**

three thematic categories emerged: Nursing training in palliative care; Potentialities for teaching palliative care; and Challenges of teaching in palliative care. The coordinators described as potentialities: transversality, theoretical and practical approach, optional subject, university extensions, interdisciplinarity and transdisciplinary approach, and as challenges: biomedical model in health education and insufficient professor training.

**Final Considerations:**

palliative care teaching in the researched institutions in the nursing education process is approached in an incipient and fragmented way, and almost always without having a specific curricular component on the subject, being present as one of its contents.

## RESUMO

**Objetivos:**

analisar a percepção dos coordenadores de curso de graduação em enfermagem sobre a formação do enfermeiro em cuidados paliativos.

**Métodos:**

estudo descritivo, de abordagem qualitativa e análise de conteúdo temática, realizado com coordenadores dos cursos de enfermagem em Instituições de Ensino Superior do Rio Grande do Norte.

**Resultados:**

emergiram três categorias temáticas: *Formação do enfermeiro em cuidados paliativos; Potencialidades para o ensino dos cuidados paliativos*; e *Desafios do ensino em cuidados paliativos*. Os coordenadores descreveram como potencialidades: transversalidade, abordagem teórica e prática, disciplina optativa, extensões universitárias, interdisciplinaridade e abordagem transdisciplinar, e como desafios: modelo biomédico na formação em saúde e formação docente insuficiente.

**Considerações Finais:**

o ensino dos cuidados paliativos nas instituições pesquisadas no processo de formação do enfermeiro é abordado de forma incipiente e fragmentado, e quase sempre sem ter um componente curricular específico sobre o tema, estando presente como um de seus conteúdos.

## RESUMEN

**Objetivos:**

analizar la percepción de los coordinadores de cursos de graduación en enfermería sobre la formación de enfermería en cuidados paliativos.

**Métodos:**

estudio descriptivo, con abordaje cualitativo y análisis de contenido temático, realizado con coordinadores de cursos de enfermería en Instituciones de Educación Superior de Rio Grande do Norte.

**Resultados:**

surgieron tres categorías temáticas: Formación de enfermeros en cuidados paliativos; Potencialidades para la enseñanza de cuidados paliativos; y Desafíos de la enseñanza en cuidados paliativos. Los coordinadores describieron como potencialidades: transversalidad, enfoque teórico y práctico, materia optativa, extensiones universitarias, interdisciplinariedad y abordaje transdisciplinar, y como desafíos: modelo biomédico en educación para la salud e insuficiente formación docente.

**Consideraciones Finales:**

la enseñanza de los cuidados paliativos en las instituciones investigadas en el proceso de formación de enfermería es abordada de forma incipiente y fragmentada, y casi siempre sin tener un componente curricular específico sobre el tema, estando presente como uno de sus contenidos.

## INTRODUCTION

Palliative care (PC) is defined as an approach that improves the quality of life of patients and their families facing problems associated with serious and potentially fatal illnesses, through the prevention and relief of suffering, early identification, accurate assessment and treatment of pain and physical, psychosocial or spiritual problems. The global need for PC will continue to increase as a result of the growing burden of chronic conditions and an aging population^(1)^.

In PC care, an essential component is multidisciplinary work, with emphasis on the work of nursing professionals, since they are the ones who most often provide greater assistance to patients in PC^(2)^. This allows them to establish an interpersonal relationship, closer and more helpful, in addition to carrying out care practices, having the opportunity to know the existential meaning of the illness.

In order to achieve nursing care in PC, it is necessary to look at the health training process. Although this assistance is a duty^(3)^, it is observed that, in general, nurses or nursing students are not sufficiently trained to deal with patients in PC^(4-5)^.

Although many undergraduate nursing courses have experienced recent curricular reformulations, it is clear that PC teaching in nursing professionals’ training has a strong influence of clinical and theoretical teaching, based on the biomedical and curative model, to the detriment of teaching more focused on coping with situations of death and dying in PC^(4,6)^, although death is part of life and is part of nursing professionals’ daily lives.

At the global level, evidence^(6-8)^ demonstrates that professional development in this area is essential for all nurses and other health professionals, as PC, in addition to scientific and technical knowledge, requires a base of human sciences, that lead them to competence and self-control in the face of the challenges to be faced, in a way that results in a greater commitment to the assistance provided as well as meeting the needs of the growing number of people with potentially fatal serious illnesses and their families.

In the literature^(4-5,9-12)^ difficulties experienced by nursing professionals and students in clinical practice in PC stand out, being cited: lack of knowledge; lack of adequate training; gap in conduct standardization; communication problems and involvement of multidisciplinary team members; little nursing participation in the decision-making process; precarious ability with religious and spiritual matters; and negative feelings such as fear, anguish, anxiety and guilt.

Given the above, it is understood to be a scenario that requires attention from educational managers to establish strategies focused on strengthening nursing training in PC, because, possibly, such difficulties encountered in this assistance are related to the training process.

## OBJECTIVES

To analyze undergraduate nursing course coordinators’ perception about nursing training in PC.

## METHODS

### Ethical aspects

The present study was approved by the Research Ethics Committee of the *Universidade Federal do Rio Grande do Norte*. The consent of the institutions involved was requested. Participants were informed about the research objectives and authorized the use of information for scientific purposes, with the signing of the Informed Consent Form. Participant anonymity was guaranteed by using pseudonyms of butterfly species, since the butterfly is the symbol of PC that, in turn, assist and comfort the process of change in patients’ and families’ lives, until the butterfly leaves its cocoon and conquers its freedom, which is a symbolic representation of the soul and body, respectively^(13)^.

### Study design

This is a descriptive study, with a qualitative approach, conducted and structured in line with the Standards for Reporting Qualitative Research (SRQR) verification instrument^(14)^. This manuscript was extracted from a master’s thesis entitled “*Formação do enfermeiro em cuidados paliativos no estado do Rio Grande do Norte*”, presented to the Graduate Program in Nursing, *Universidade Federal do Rio Grande do Norte*.

### Study setting and participants

The study was carried out in Higher Education Institutions (HEIs) located in the state of Rio Grande do Norte. These HEIs were identified through an online search in the e-MEC System, resulting in a quantity of 19 HEIs. Of these, six were excluded because they did not yet have an undergraduate nursing class by the period established for data collection (August to October 2018) and another two, whose representatives refused to participate. Therefore, 11 HEIs in Rio Grande do Norte with an undergraduate nursing course were included, with graduating and active classes in 2018. Among these, seven are located in the capital, six are private and one is public, and the others, four, are located in other municipalities in the state and are public.

The sample was of the intentional type and its population included representatives of the selected HEIs’ course coordination. Course coordinator - titular or deputy -, with official performance for at least three months, were included. Coordinators on leave during the collection period or who had conflicts of interest as collaborators in the research were excluded. Each HEI had a course coordinator, which represented 11 participants, and, of these, seven had deputy coordinators. In the end, 18 participants met the inclusion criteria. Interviews were prioritized with the HEI’s head coordinator. After applying the exclusion criteria, two main coordinators were excluded, and therefore, an interview was carried out with their respective vice-coordinators. Thus, 11 participants participated in the study, nine of which were senior coordinators and two deputy coordinators.

### Data collection

The semi-structured interview technique was used, guided by a form containing questions related to participants’ personal and professional information, the profile of graduated nurses, approach to PC in the undergraduate course and the methods or strategies adopted in the teaching-learning process about PC. Interviews were carried out by the main researcher, between August and October 2018, after prior contact and according to the participant’s availability, in a comfortable and reserved place at HEIs. Audio was recorded on an MP4 device and transcribed in full. In order to carry out some interviews, it was necessary for the researcher to travel to other municipalities.

### Data analysis

The data analysis used was thematic content analysis^(15)^, structured by Minayo^(16)^, according to the following steps: pre-analysis; material exploration; and treatment of results/inference/interpretation. A comprehensive reading was carried out of the set of material selected and obtained in all audio interviews that were exhaustively recorded. Then, material exploration consisted of: 1) distribution of excerpts; 2) reading, dialoguing with the parts of analysis texts; 3) identification of the nuclei of meaning pointed out by the parts of the texts; 4) dialogue with perhaps the nuclei of meaning and initial assumptions; 5) analysis of the different nuclei of meaning in search of broader themes; 6) regrouping parts of the texts by themes found; and 7) elaboration of an essay by theme, in order to account for the meanings of the texts and their articulation with the concepts that guide the analysis, interrelating them with the results obtained, data from other studies and theoretical concepts^(16)^.

From the analysis, four thematic categories emerged and, to contemplate the objective of this manuscript, a clipping was carried out with discussion referring to the three main thematic categories, presented below. During the process of analysis and reduction of the text to meaningful words and expressions, these categories made it possible to construct subcategories.

The results achieved are presented and discussed according to the theoretical foundation of complex thinking proposed by Edgard Morin^(17)^, in addition to comparison with the updated scientific literature on the subject.

Choosing the systematized complex thought of philosopher Edgar Morin as a theoretical framework was due to his dealing with issues involving education based on a perspective of change and with the aim of developing reflective subjects about themselves and the world, based on criticism and transforming reflection. Moreover, it contributes to a better understanding of the phenomena related to PC, as it is a broad topic in its complexity and understands man as an example of complexity.

The challenge of complexity is based on the need to reconnect what is usually separated, to contextualize knowledge and to provide interaction between certainty and uncertainty. Thus, what is complex needs to capture interrelationships, different and conflicting realities, through thinking that respects diversity, seeking to unite rather than simplify^(18)^.

Morin brings seven principles that help to understand complex thinking, they are: organizational recursion; holographic; retroactive loop; circular recursive movement; autonomy/dependence; dialogic; reintroduction of knowledge in all knowledge^(17)^. Of these, organizational recursion, holographic and dialogical are the three fundamental principles^(18)^ and provided a theoretical basis for the study.

## RESULTS

The participants of this study correspond to a total of nine titular coordinators and two deputy coordinators of undergraduate nursing courses in the state of Rio Grande do Norte. Of the 11 participants, six were female and five were male; age ranged from 27 to 54 years; most with married marital status; and all of them graduated in nursing, with time to complete the course varying between five and 30 years.

All participants performed, in addition to the role of course coordinator, teaching activities, with time in service at the institution ranging from nine months to 15 years, and the majority had more than 12 years in the institutions. It was verified that six of these participants were in their first term, with a time of performance from three months to four years, and the others, that is, five participants, were already in their second term, therefore, with more than four years in the function of course coordinator. Regarding the level of education, four had a doctorate, another four had a master’s degree, and three had a specialization. Only three participants stated that they had already taken training courses on the subject of the study.

After the material exploration phase, three thematic categories that emerged from study participants’ speeches were analyzed, being presented in the following order: *Nursing training in palliative care; Potentialities for teaching palliative care*; and *Challenges of teaching in palliative care*.

### Category 1 - Nursing training in palliative care

This category addresses, in essence, elements and characteristics of PC teaching, and gave rise to the following two subcategories: *Palliative care in undergraduate nursing teaching*; and *Insufficient education in palliative care*.

#### Palliative care in undergraduate nursing teaching

This subcategory shows, from the perspective of course coordinators, how PC is presented in undergraduate nursing education. For most interviewees, this is a content offered within other curricular components, according to the following statements:

*We do not have a specific subject in palliative care. They are contents discussed in the course, during the course.* [...] *we work palliative care within the life cycle.* (Red Admiral Butterfly)

*We have the thanatology subject, adult health subjects, we have elective subjects such as cancer patient care.* [...] *it turns out that all these subjects directly or indirectly address this content.* (Queen Alexandra’s Birdwing)

*I believe that the subject of oncology or elder health says something about this, but not related to palliative care, but about the process of death and dying, the issue of older adults who are sick and do not have an extra life time. And if it is treated, it’s more in theory.* (Swallowtail Butterfly)

[...] *we work within these subjects that contemplate adult health, where we see ICU, emergency room, medical clinic, surgical center, ethics and, then, within this content, we also contemplate palliative care.* (Leafwing Butterfly)

[...] *it is mainly worked in the subject of adult health.* (Owl Butterfly)

*Saying that there is a specific subject does not exist, it is widespread in contexts.* (Pieridae Butterfly)

In addition to the theoretical approach disseminated in the subjects, there is an increase in PC during practical curricular activities experienced by students during graduation in the hospital setting or in Primary Health Care. Experiences in extracurricular internships in highly complex oncology centers are also mentioned. However, it can be said that there are still no specific fields of clinical teaching in undergraduate nursing for the theoretical-practical approach to PC, considering that all students will experience these experiences.

*So, the methodology goes beyond theoretical classes.* [...] *we have practical interventions, practical interventions they take place, in relation to palliative care more in the hospital field and at home.* [...] *At the hospital he* [student of eighth and ninth period] *rotates through the different sectors, surgical center, emergency, in the clinics, Intensive Care Unit. At these times, he works directly with individuals who may be in palliative care. They have contact with terminally ill patients or those who need palliative care, for instance, in home visits, monitoring bedridden patients, or in some more critical cases at the hospital level.* (Julia Butterfly)

*So, if we have it here in theory, it will have it almost in practice, I won’t say that it will always have it, for instance, there’s no way in the emergency room, right? Semi-technical, for instance, but there are subjects that you can work with, how to have a more observatory perception.* (Pieridae Butterfly)

*We know that we will not necessarily have this experience in all practices.* (Queen Alexandra’s Birdwing)

*Perhaps our student who is in practice, in the final stage of the course, has primary care contact with this type of patient.* (Leafwing Butterfly)

[...] *we have students who are in centers of high complexity of oncology, which is a place where people arrive with an irreversible diagnosis of cancer, and it is where they have a greater shock.* [...] *remembering that not all students go to this center, it’s just a part, when a student goes through a selection process, and so not all. So, we cannot say that everyone goes through this experience.* (Morpho Butterfly)

#### Insufficient education in palliative care

It can be said that, given the population’s current health needs, there is little focus on PC in professional nursing education and training in the researched HEIs. From the theoretical approach, with minimal transversal content throughout the curriculum, to practical teaching, where PC experiences are occasionally carried out, without prior planning with objectives to be achieved. This context is recognized in the following statements:

*We do not have a specific unit on palliative care, and this is a gap that we have. Whenever we are evaluating the curriculum, we find these needs, due to the breadth of health needs.* (Julia Butterfly)

*Palliative care is something that is being addressed now, and in a very limited way. I believe that this is not an exclusive experience of this institution. Evolution is necessary, considering the epidemiological information* [...]. *Students do not need to leave as specialists, but they need to leave with knowledge about, and I believe that knowledge about is still incipient.* (Sylphina Angel Butterfly)

*In graduation, palliative care is very lacking, the reflection of that is us, and that is not my institution* [...]. *I understand that this theme was not so necessary 50 years ago, today it is because the content must accompany the change of a society.* [...] *we leave graduation as if we were wearing clothes, and our clothes are full of holes, some bigger holes and other smaller holes. Some holes because the training didn’t offer you and others because you didn’t close them as a student, some holes are smaller and some are bigger. Today, the hole caused by the absence of palliative care is a big hole.* (Emerald Swallowtail Butterfly)

### Category 2 - Potentialities for teaching palliative care

It is a category that originated six subcategories: *Palliative care as a transversal theme; Theoretical and practical palliative care teaching; Conception of optional subject in palliative care; University extension in teaching palliative care; Interdisciplinarity and transdisciplinary approach in teaching palliative care; Nursing as a potential to develop palliative care*.

#### Palliative care as a transversal theme

According to some of the coordinators, PC can be approached in nursing education from the first training periods, from a more general approach, becoming more complex during graduation.

*Although we understand that palliative care is a transversal theme. This content must not be tied to a subject.* (Emerald Swallowtail Butterfly)

*I imagine that this theme would need to be dealt with in a transversal way, in all the subjects that could, from the beginning of the course.* [...] *and with the advancing complexity of the subjects, it could be treated in a more specific way.* (Apollo Butterfly)

*So, I believe that we really have to think about transversality and introduce these themes that are more complex, in a primary, initial way, in all subjects that are open to this.* [...] *we can have this palliative care being introduced in a lighter way, until we reach the most complex way of being.* (Queen Alexandra’s Birdwing)

There is a tendency for some interviewees to focus on PC as transversal knowledge, addressed throughout the entire curriculum structure. However, at the same time, fear that, in this way, there will be uncontrolled freedom over the contents in its components, and this will cause a poorly organized implementation of the theme by professors, as shown in the following speech:

*I bet on transversality, but I am also afraid* [...] *my fear is to put palliative care as a transversal theme in all subjects, it will be up to the professor to include it, and it is risky.* (Apollo Butterfly)

It is perceived the need for the theoretical principles that support the philosophy of PC to be present in a transversal way in the undergraduate curricula during nursing training, with a view to the training process’ continuous learning.

*And if all the components that have this transversality need to be focused as a component, we often get lost with many subjects that do not account for the whole.* [...] *this is the big problem, it is always having other components, but again fragmenting the training of professionals and not contributing to thinking about the complexity of human beings.* (Morpho Butterfly)

[...] *it can be transversally present during training* [...] *because isolating in a single subject can be reductionist and it does not account for reality.* (Julia Butterfly)

#### Theoretical and practical palliative care teaching

For the teaching-learning process of PC, coordinators suggested that the approach in theoretical teaching be sequenced by the context of practical teaching, through clinical immersions, in mandatory internships or practical classes in real and uncontrolled scenarios, as idealized by the speeches:

*The best way to approach this is in practice.* [...] *it is at that moment when the individual discovers that caring is not just caring for a physical body, a specific disease, but it is caring for a human being, someone who has several relationships.* [...] *that’s when a certain attitude, a certain conduct in the face of this process is born.* [...] *it is the professor’s opportunity to discuss: what other forms of care do we have? What can we at this moment provide in terms of well-being to the other? By experiencing practice, they manage to make connections with the theory and then rescue the experience that students have in the hospital and bring it up for discussion, to give a new meaning to that.* (Morpho Butterfly)

*So, I think the best strategy would be this, we bring knowledge into the classroom and take him to have this experience, this practice so that he can really have contact with this problem in the service.* (Owl Butterfly)

[...] *the ideal would be to rotate this student to the real field, where they will really have knowledge of the situation, in the final stoning of this student, they need us to develop scenarios with cases* [...]. (Emerald Swallowtail Butterfly)

#### Conception of optional subject in palliative care

Other reports also showed the possibility of designing an optional course to approach PC:

[...] *I believe that we could have an optional subject, since things need to be added over time.* (Sylphina Angel Butterfly)

[...] *this content, we want to work more, including offering an optional subject.* (Leafwing Butterfly)

The creation of an optional subject is an alternative that can overcome possible gaps in nursing training and respond to the population’s current health needs, but it cannot cover and generate interest in the subject for all undergraduates, as evidenced by the following speech:

*The inclusion of an optional subject is being discussed, but I don’t know if it will have an impact, because, because it is optional, some students will take it, others will not. So, the graduate who did not attend will leave with weaknesses.* (Apollo Butterfly)

#### University extension in teaching palliative care

This subcategory refers to other results found among the statements that also showed the possibility of university extension activities, being cited as a strategy in PC teaching for nursing training, because the integration of health services with HEIs can provide benefits for both and advance knowledge in PC, according to the following statement:

*The university extension would be one of the ways that we could get closer to the service.* [...] *and create this exchange between the institution and the university, and thus contribute to improving the service.* [...] *and I believe that the extension can be a door for us professors to appropriate more of the knowledge and then it can flow better within the university itself.* (Owl Butterfly)

#### Interdisciplinarity and transdisciplinary approach in teaching palliative care

Another possibility present in coordinators’ speeches is related to the importance of interdisciplinarity and transdisciplinarity in PC teaching, with the creation of educational opportunities in which students from different areas of health acquire the necessary skills for patient-centered practice:

*The training process could have concomitant subjects with all multidisciplinary team members* [...]. (Sylphina Angel Butterfly)

[...] *the comprehensive is taking care of a whole that is within a whole, because a person who leaves will leave social, economic, psychological consequences, their history within a society.* [...] *nursing graduates need to leave with this ingrained knowledge, that they need to take care of this subject, make him live well until the last moment. However, he will not be able to do this alone, he needs a psychologist, social worker, doctor, physiotherapist, because, to give him conditions to live well, he needs a multidisciplinary team, nursing alone is not enough, he also needs the family, so he needs a whole set* [...]. (Red Admiral Butterfly)

[...] *palliative care should be available to everyone, starting with the fact that you have to work in a multidisciplinary, interprofessional way. But we don’t have that reality, who knows in the near future. I think that for students it is essential.* (Pieridae Butterfly)

#### Nursing as a potential to develop palliative care

The coordinating participants recognized that, among the health professions, nursing has the potential to develop PC, considering its position of greater proximity in care.

[...] *nurses are a fundamental and main actor in palliative care, often medical attention or that of other professionals is restricted to specific moments and who will continuously monitor patients in palliative care are nurses.* (Apollo Butterfly)

[...] *nursing works directly with this part of palliative care.* [...] *nursing deals directly with this patient, especially in oncology.* (Swallowtail Butterfly)

[...] *because it is our essence, we will deal with it daily* [...]. (Morpho Butterfly)

### Category 3 - Challenges of teaching in palliative care

This third thematic category shows, according to the results issued by interviewees, the challenges/obstacles that are announced in the insertion of this content in PC teaching in nursing training, and originated the two following subcategories: *Biomedical model in health training*; and *Insufficient professor training in palliative care*.

#### Biomedical model in health education

With regard to this subcategory, Morpho butterfly’s speech reflects that health training is part of a positivist and curative logic, and that in a way, with PC, it presents a counterpoint, in the face of the more complex context:

*Our training does not leave us much opportunity to think about these questions. We think in a predominantly positivist way* [...] *of great limitation in the area of health. We need to be a little more aware of our limits.* [...] *This training needs to change, even more in the sense of thinking about the human being in a more complex way, we are still very attached to this issue of the disease, in believing that care involves a cure.* (Morpho Butterfly)

#### Insufficient professor training in palliative care

This subcategory signals a certain distance and difficulties on the part of professors between theoretical and practical teaching in PC as well as the existing barriers to carrying out training and meet the deficits in professors’ knowledge so that it can fill this gap, as corroborated by the following statements:

[...] *we don’t have training in the area and we don’t even have the confidence to approach the theme within the subjects.* [...] *the teaching staff needs to be qualified for this generation of knowledge.* [...] *the professors who are in the classroom today experienced the nursing of yesterday, and the nursing of yesterday did not prepare us for that.* [...] *we cannot bring to the classroom what we have not understood or learned. And we have not sought, for various reasons, training is not cheap for palliative care.* (Emerald Swallowtail Butterfly)

[...] *professors need to be trained on the subject. I’m not sure if the professors who work directly in these fields* [...] *are qualified for this, I’m not particularly.* (Apollo Butterfly)

[...] *Yes, there is a need for specific training so that we don’t learn by doing. It would be interesting if the courses started to have people with specific training in this subject* [...]. (Julia Butterfly)

## DISCUSSION

Professional practice, in the context of PC, proves to be fundamental and a necessary area of learning in nursing education, when considering the population’s current health profiles. However, the results of this study reveal an absence of specific subjects and fields of clinical teaching in undergraduate nursing to approach theoretical-practical teaching in PC, and this may be compromising nurses’ professional training. Similar results were confirmed in national and international literature^(2,4,9,19)^.

It is noted that the lack of teaching in PC represents one of the main barriers for the population’s access to these services, given the deficit of qualified professionals who could contribute to improving the service of a repressed demand. Thus, in an effort to improve PC based on nursing training on the subject, the End-of-Life Nursing Education Consortium (ELNEC) project was created in the United States in 2000, which represents a collaboration between the City of Hope and American Association of Colleges of Nursing^(19)^, which has been used around the world to improve the quality of end-of-life care and nurses’ views and attitudes towards end-of-life care.

The results of this study show that the PC theme is present in nursing education, mainly through curricular components that address adult and elder health, ethics and bioethics, and, in particular, in oncology subjects.

In Brazil, among HEIs that provide some subject for studies and/or reflection on PC, most are still optional. In addition, there is an unequal distribution in the regions of the country in relation to the offer of this education, as what happens, for instance, in the Northeast and Southeast, regions with the greatest supply of components on PC, when compared to the North, Midwest and South^(2)^.

Even identifying the gaps in PC teaching in nursing training in the context of this study, it is perceived that there is potential for the creation of this teaching, such as transversal teaching, theoretical and practical teaching experiences, conception of optional subject, university extensions, interdisciplinarity and transdisciplinary approach, and nursing as a profession with potential to develop teaching in PC.

Corroborating the results of this study, the literature recommends including obligatory subjects to introduce PC from the early years of the course and recommends having content on its history, philosophy and guiding principles in its structure. And in later stages, it is recommended to offer specific subjects on nursing care in PC, with practical components, to guarantee students’ access to information and experiences, non-pharmacological therapies, communication, interdisciplinary care and quality of life promotion. It is also considered of extreme importance that contents about the process of dying are addressed in mandatory subject^(20)^.

Based on complex thinking, regarding Morin’s principle of organizational recursion, which consists of uniting the knowledge of the parts to the knowledge of the whole^(17)^, the importance of transversality and interdisciplinarity is highlighted as potentialities for PC teaching.

Interdisciplinarity is understood as exchange and cooperation between subjects. From this perspective, the education system reform should encourage the ability to contextualize and integrate rather than overvaluing abstraction and formalization^(17)^. Thus, training is aimed at uniting and integrating physical, psychological and spiritual aspects in patient care, through interdisciplinary teams, fundamental to PC.

Based on the second principle, holographic, it is possible to see men in order to contextualize them, to see them inserted in the world as a tiny part in the whole, but containing the presence of the whole in the part^(17)^. It is also based on this conception that health training in the context of PC needs to be aligned. When assisting patients in PC, issues intrinsic to the human condition are covered, such as subjectivity of pain, the meaning of suffering/forgiveness, cultural diversity, ethics, individuality of people being cared for and their emotional, psychological, political, social, spiritual issues, among others, which are interrelated in continuous interactions in contexts of this nature.

Among the possible facilities for learning PC in clinical practice, research carried out with Portuguese and Brazilian students identified the support provided by professors and other professionals in relation to previously acquired knowledge in PC and interprofessional care^(20)^.

It is understood that there are many challenges present in PC teaching in nursing education, and one of them refers to the biomedical model overvaluation, always evidenced by other studies^(20)^. In fact, there is an overvaluation of the curative model in health education, with the study of pathologies aimed solely at cure; however, this educational concept requires reformulation, with a view to meeting human needs in their entirety^(2)^.

In this sense, reinforcing Morin’s organizational and holographic principles of recursion, it is emphasized that the world is seen in its entirety, interconnected, and not as the sum of separate parts. Therefore, complexity integrates the way of thinking and opposes the reduction of parts or the mechanism of Cartesian thinking. It is based on this distinction - training from a complex perspective versus Cartesian thinking - that one can understand the dialogical principle, responsible for associating two terms that are complementary and antagonistic at the same time, and rationally assume the association of contradictory notions to conceive the same complex phenomenon^(17)^.

For this reason and in this context, Morin questions the way education has been constructed, based on a paradigm of positivist science that, by establishing a unique relationship of cause and effect, reduces, separates and simplifies knowledge. There is an inadequacy between school knowledge, separated into subjects, and that the problems and challenges of humanity are complex, transdisciplinary, multidimensional, global and planetary^(17)^.

In another challenge presented by the educational managers in this study, there is insufficient professor training in PC teaching. For them, there is concern about how to transmit knowledge within a hierarchical social structure, but at the same time, in permanent transformation. It is recognized that it is a great challenge to deal with new knowledge required by modern society, which will contribute to new knowledge in the education of the future. To do so, it is necessary to reform thinking, articulate and organize knowledge and, thus, recognize and understand the world’s problems^(21)^.

Thus, based on this understanding, it can be seen that isolated and successful experiences have been analyzed all over the world, being in line with the potential identified in this study, and serving as an inspiration for teaching PC in nursing education. For instance, a study^(22)^ is cited that describes a successful experience of implementing a pioneering and innovative curriculum, which transmits knowledge in PC transversally, through theory and clinical practice in health environments, culminating in in their training with the completion of a compulsory subject in the fourth year of the course.

The teaching modality, via academic extension, was also mentioned with enthusiasm and potential for teaching in PC. In this perspective, it is believed that the participation of undergraduate nursing students in projects of this nature favors the formation of subjects capable of recognizing and intervening in real problems, in search of social transformations that liberate and transform their field of action^(23)^. According to Morin, knowledge progresses not by sophistication, formalization and abstraction, but, above all, by the ability to contextualize and encompass. Thus, relevant knowledge is able to place any information in its context^(17)^.

In line with this reflection, Morin highlights in “a well-made head” the meaning that, instead of accumulating knowledge, the most important thing is to have a general aptitude for posing and dealing with problems, linking knowledge and giving them meaning. Therefore, education must favor the mind’s natural aptitude to pose and solve problems. Therefore, it is necessary to encourage and instigate the interrogative capacity and guide it towards the fundamental problems of our time^(17)^.

Evidence^(19,24-25)^ confirms the benefits of PC education such as: significant improvements related to subject knowledge and understanding; attitude and confidence; ruptures and the conception of new ways of thinking and caring in line with PC principles; development of skills and attitudes inherent to PC; perception of the importance of interdisciplinarity, emotional balance, humanization of care, reflections about life and death and personal values.

### Study limitations

The researched scenario is highlighted as a limitation, and perhaps its results cannot be generalized to all nursing HEIs in the country. Another limitation was the investigated population having only nursing coordinators (even though all of them are also professors), not including other professors and students involved in the teaching-learning process in undergraduate nursing courses. It stands out, in the literary investigations carried out, the absence of studies that have carried out this type of research with nursing coordinators, being perhaps as an innovator in the nursing education area.

### Contributions to nursing, health or public policies

This study contributes to fostering the necessary transformations in PC teaching in the nursing training process, especially in the researched context, directing towards more investments in research, debates, suggestions for teaching the theme that are capable of changing the identified reality given the current importance of the topic.

## FINAL CONSIDERATIONS

The theme about PC is still approached in an incipient and fragmented way in the training of generalist nurses, and sometimes it is present only as one of the contents within other curricular components. This is a reality emitted by all nursing course coordinators surveyed and which signals a challenge to be pursued by reformulators and managers of nursing curricula, given the current epidemiological profile that is presented in Brazil.

Edgar Morin’s complex thinking can contribute to nursing training in PC, as he understands man as a complex being and articulates all knowledge (reconnection of knowledge), inherent to the health training process and the PC scenario.

As a recommendation, it is suggested to insert the content transversally throughout nursing training, associated with practical experiences in real and uncontrolled scenarios as well as the creation of specific curricular components, research and university extension activities and practical activities with an emphasis on interdisciplinarity, which consider in their approaches the essence of care inherent in the profession.
